# *Hedychium putaoense* (Zingiberaceae), a new species from Putao, Kachin State, Northern Myanmar

**DOI:** 10.3897/phytokeys.94.22065

**Published:** 2018-01-29

**Authors:** Hong-Bo Ding, Bin Yang, Shi-Shun Zhou, Ren Li, Mya Bhone Maw, Win Maung Kyaw, Yun-Hong Tan

**Affiliations:** 1 Southeast Asia Biodiversity Research Institute, Chinese Academy of Sciences, Yezin, Nay Pyi Taw 05282, Myanmar; 2 Centre for Integrative Conservation, Xishuangbanna Tropical Botanical Garden, Chinese Academy of Sciences, Menglun, Mengla, Yunnan 666303, P.R. China; 3 Forest Research Institute, Forest Department, Ministry of Environmental Conservation and Forestry, Yezin, Nay Pyi Taw 05282, Myanmar

**Keywords:** *Hedychium*, Myanmar, Taxonomy, Morphology, Zingiberaceae

## Abstract

*Hedychium putaoense* Y.H. Tan & H.B. Ding, a new species of Zingiberaceae from Putao, Kachin state, Northern Myanmar, is described and illustrated. It is similar to *H. densiflorum* Wall. and *H. longipedunculatum* A.R.K. Sastry & D.M. Verma, but differs by its very small bract (4–6 × 2.5–3 mm vs. 18–19 × 5–5.5 mm and ca. 11 × 7 mm, respectively), semicircle and dark red bracteole, orange flower and broadly falcate to lanceolate lateral staminodes.

## Introduction

The pantropical Zingiberaceae is the largest family in the monophyletic order Zingiberales with 53 genera and more than 1377 species (Kress et al. 2002; Pederson 2004; Kong et al. 2010). *Hedychium* Koenig, commonly called the “ginger lily” or “butterﬂy lily”, produces one of the most beautiful and fragrant flowers in the family Zingiberaceae (Sanoj et al. 2010). The genus was established by Koenig in 1783, based on the species *H. coronarium* Koenig. There is currently little consensus on the number of species, with recent estimates varying from about 50 (Wu and Larsen 2000) to 80 (Sirirugsa and Larsen 1995) and these are mainly distributed throughout most of tropical Asia (Sirirugsa and Larsen 1995; Wood et al. 2000). The genus has its highest species diversity in the tropical and subtropical Himalayan region (Sanoj et al. 2010). *Hedychium* is characterised by flowers with very long (rarely short) filaments, dorsifixed anther and usually fragrant flowers (Hu and Liu 2010a). Members of the genus can be easily recognised by their showy, many-flowered terminal spikes, some of which have been cultivated worldwide (Picheansoonthon and Wongsuwan 2011).

Several new species of *Hedychium* have been described in the last few decades (Williams et al. 2003; Wongsuwan 2008; Picheansoonthon and Wongsuwan 2009, 2011; Sanoj et al. 2010; Hu and Liu 2010a, 2010b; Sanoj and Sabu 2011; Thomas et al. 2015; Odyuo and Roy 2017). So far, 15 species of *Hedychium* have been recorded in Myanmar: *H. bordelonianum* W.J. Kress & K.J. Williams, *H. coccineum* Buch.-Ham. ex Sm., *H. coronarium* Koenig, *H. elatum* R. Br., *H. ellipticum* Buch.-Ham., *H. flavum* Roxb., *H. forrestii* Diels, *H. gomezianum* Wall., *H. gracile* Roxb., *H. marginatum* C.B. Clarke, *H. spicatum* Sm., *H. stenopetalum* Lodd., *H. thyrsiforme* Sm., *H. venustum* Wight, and *H. villosum* Wall. (Kress et al. 2003).

From April to May in 2017, a team from the Xishuangbanna Tropical Botanical Garden (XTBG) in collaboration with the Forest Research Institute of Myanmar, conducted field work in Northern Myanmar to survey plant diversity. During field work, some interesting specimens of *Hedychium* were found in Putao, Kachin state. Based on a detailed examination of the morphological characteristics of the collected material and those of the closely related similar species, the authors have arrived at the conclusion that the specimens of *Hedychium* collected in Myanmar belong to a species new to science, which are described and illustrated herein.

## Material and methods

Measurements and morphological character assessments of the new species *Hedychium putaoense* have been examined based on fresh materials and dried specimens. It has been compared with the morphologically similar species *H. densiflorum*, *H. longipedunculatum*, with affinities inferred using descriptions, type specimens and other herbarium specimens (Wallich 1832; Sastry and Verma 1968; Wu and Larsen 2000; Moaakum and Dey 2013). Protologues and images of type specimens were gathered from JSTOR Global Plants (http://plants.jstor.org).

## Taxonomic treatment

### Hedychium putaoense

Taxon classificationPlantaeZingiberalesZingiberaceae

Y.H.Tan & H.B.Ding sp. nov.

urn:lsid:ipni.org:names:77175483-1

Figure 1

#### Diagnosis.

*Hedychium putaoense* Y.H. Tan & H.B. Ding is morphologically similar to *H. densiflorum* Wall. and *H. longipedunculatum* A.R.K. Sastry & D.M. Verma, but it can be easily distinguished by its very small bract (4–6 × 2.5–3 mm vs. 18–19 × 5–5.5 mm and ca. 11 × 7 mm, respectively) and bracteole (2–2.5 × 3–3.5 mm vs. ca. 9 × 2 mm and ca. 6 × 4 mm, respectively), orange flower and broadly falcate to lanceolate lateral staminodes.

#### Type.

MYANMAR. Kachin State: Putao District, the top of the mountain from Masabu village to Namti village. Epiphytic herbs in tropical montane forests, 97°17'42"E, 27°25'29"N. 1700 m a.s.l., 13 May 2017, flowering, *Myanmar Exped. M1724* (holotype: HITBC!; isotypes: HITBC!)

#### Description.

Epiphytic, sometimes terrestrial, perennial rhizomatous herbs, light greyish-green externally. Leafy shoot 20–50 cm high, slanting with erect inflorescence. Leaves 4, spreading, sessile, upper petioled; petiole 2–10 mm long; ligule ca. 8–19 mm long, apex obtuse, glabrous, membranous, translucent, reddish brown when fresh, yellowish brown when dry, closely appressed to the stem; lamina 13–23 × 3–7 cm, lanceolate, dark green above, pale green below, or sometimes light purple-tinged below, glabrous; margin undulate, membranous, non-ciliate; apex narrowly caudate, twisted, base attenuate. Inflorescences 7–10 cm long, cylindrical, dense, erect, rachis glabrescent. Bracts 4–6 × 2.5–3.0 mm, ovate, purplish-red, glabrous, convolute, margin ciliate, membranous, tip acute, cincinnus 1-flowered. Bracteoles 2.0–2.5 × 3.0–3.5 mm, semicircular, dark red, glabrous, membranous, acute at tip, margin ciliate. Flower 2.8–3.9 cm long, small, orange, nearly entire inflorescence open at a time, ascending. Calyx 1.2–1.4 cm long, 2.0–2.5 mm wide at mouth, tubular, pale light red, densely villous, tufted hairs at tip, membranous, upper half inflated, lower part closely appressed to corolla tube, unilaterally split up to ca. 5 mm depth. Corolla tube 10–13 mm long, creamy yellow, glabrous, tube intensely curved towards tip about 90° to 180°. Corolla lobes 10–16 × 1–2 mm linear-lanceolate, red, membranous, glabrous. Lateral staminodes 11–13 × 3–4 mm, broadly falcate to lanceolate, clawed towards base, orange, spreading on flower, obtuse at tip, intensely reflexed back. Labellum 12–14 × 4–7 mm, widely obovate, orange, spreading on flower, base cuneate, sinus ca. 2–3 mm deep, lobes oblong, obtuse at tip. Stamen 1.4–1.7 cm long. Filament 1.1–1.2 cm long, ca. 2 mm wide at base, orange, creamy yellow towards base, intensely curved towards tip about 180°. Anthers ca. 5 × 2 mm, oblong, orange, split opens from top to bottom, attached with the filament at ca. 1 mm above from base, thecae parallel with the filament; connective red, glabrous, non-crested. Ovary ca. 2.5 mm diameter, sub-globose, densely villous. Style filiform, creamy white, glabrous, dark red-tinged towards stigma. Stigma ca. 0.5 mm wide, dark red, cup-shaped, mouth margin ciliate, ca. 1 mm exserted from the anther. Epigynous glands 2, ca. 1 mm long, oblong, orange. Fruit unknown.

**Figure 1.**
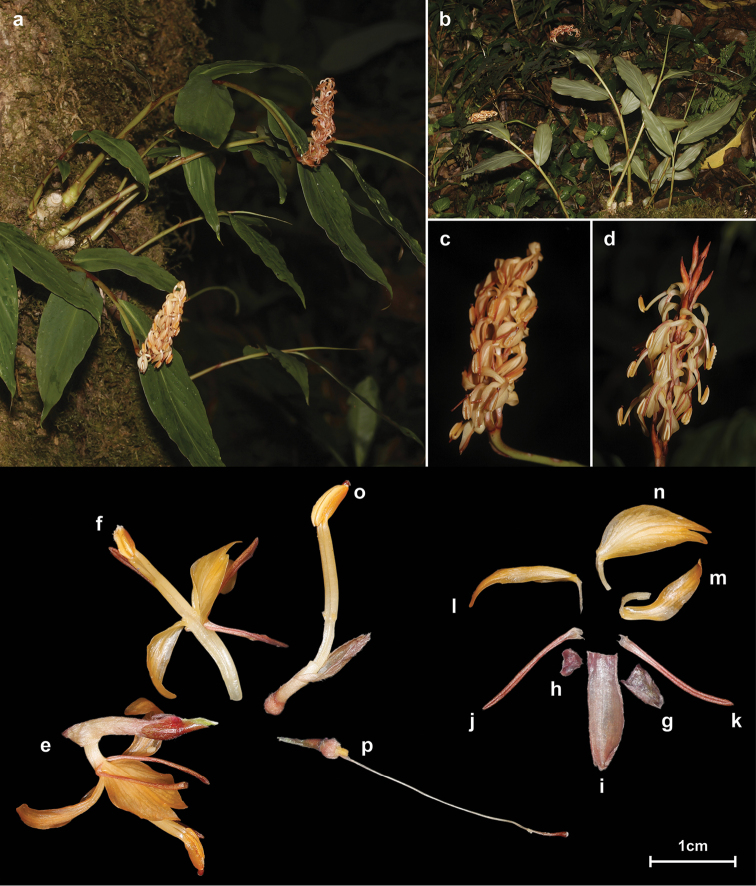
*Hedychium putaoense* Y.H. Tan & H.B. Ding. **a–b** Habit **c–d** Inflorescence **e–f** Front and lateral view of flower **g** Bract **h** Bracteole **i** Calyx **j–k** Corolla lobe **l–m** Lateral staminodes **n** Labellum **o** Corolla tube with anther and calyx **p** Ovary with pistil and glands. Photographed by Y.H. Tan & H.B. Ding.

#### Phenology.

Flowering from May to July.

#### Distribution and habitat.

This new species is known to grow at the top of the mountain from Masabu village to Namti village, Putao District, Kachin State, where it grows epiphytically on the trees of tropical montane forests at an elevation of ca. 1400–1800 m.

#### Etymology.

The species is named after the type locality, Putao county, in Kachin State, Myanmar.

#### Affinities.

*Hedychium putaoense* Y.H. Tan & H.B. Ding shares certain characteristics with *H. densiflorum* (Wallich 1832) and *H. longipedunculatum* (Sastry and Verma 1968), e.g. non-imbricating bracts, cincinnus 1-flowered, small and dense flower, oblong anther and sub-globose ovary. After comparison with specimens and descriptions in literature, it was found that *H. putaoense* can be clearly differentiated from the latter two species, even on the basis of their vegetative characters: e.g. the proportion of the bract to the calyx; *H. putaoense* has very small bracts that are shorter than the calyx (4–6 vs. 12–14 mm), whereas, the bracts of *H. densiflorum* are longer than the calyx (18–19 vs. 13–14 mm) and the bracts of *H. longipedunculatum* are equal in length with that of the calyx (ca. 11 vs. 11 mm). *H. putaoense*, furthermore, differs in having semicircular and dark red bracteole, corolla tube curved towards the tip by about 90° to 180°, orange lateral staminodes and labellum, filament intensely curved towards the tip by about 180°, densely villous ovary and dark red stigma. *H. densiflorum* has tubular and pale green bracteole, corolla tube slightly bent to one side towards the tip, orange-red lateral staminodes and labellum, straight filament, glabrous ovary and yellow stigma. *H. longipedunculatum* from India has notched ligule, hairy rachis, triangular bract, ovate bracteole, creamy yellow flower, erect corolla tube, straight filament and green stigma. A detailed comparison of the morphological differences amongst these taxa is given in Table 1 and the evidence from morphological analysis supports the recognition of *H. putaoense* as a distinct species.

**Table 1.** Comparison of key morphological characters of *Hedychium putaoense*, *H. densiflorum*, and *H. longipedunculatum*.

Attributes	*H. putaoense*	*H. densiflorum*	*H. longipedunculatum*
Ligule	8-19 mm long apex obtuse	10–11 mm long apex obtuse	15–20 mm long notched at tip
Lamina	13–23 × 3–7 cm lanceolate	28–31 × 4.5–5.8 cm elliptic	15–23 × 6–10 cm broadly elliptic
Inflorescence	7–10 cm long rachis glabrescent	11–17 cm long rachis glabrescent	9–20 cm long rachis hairy
Bract	4–6 × 2.5–3 mm ovate, purplish red	18–19 × 5–5.5 mm elliptic, pale green	ca. 11 × 7 mm triangular, pinkish-red
Bracteole	2.0–2.5 × 3–3.5 mm semicircle, dark red	8–10 × ca. 2 mm tubular, pale green	ca. 6 × 4 mm ovate, red
Flower	2.8–3.9 cm long, orange	2.8–3 cm long, orange-red	3–3.3 cm long, creamy yellow
Calyx	12–14 mm long, pale light red	13–14 mm long, white	ca. 11 mm long, pale yellow
Corolla tube	10–13 mm long creamy yellow curved towards tip about 90° to 180°	18–19 mm long white, orange-red towards tip bent to one side towards tip	ca. 11 mm long creamy yellow with pale red erect
Corolla lobes	10–16 × 1–2 mm linear-lanceolate, red	12–13 × ca. 3 mm elliptic, yellow	ca. 16 × 3 mm linear-lanceolate, creamy yellow
Labellum	12–14 × 4–7 mm, widely obovate, orange, sinus 2–3 mm deep	9–10 × ca. 8 mm, widely obovate, orange-red, sinus 3.5–4 mm deep	ca. 15 × 6 mm, spatulate, creamy yellow, sinus ca. 1 mm deep
Lateral staminodes	11–13 × 3–4 mm broadly falcate to lanceolate, orange	8–8.5 × 5 mm elliptic, orange-red	17 × 7 mm spatulate, creamy-yellow
Stamen	1.4–1.7cm long	1.4–1.5 cm long	ca. 2.2 cm long
Filament	11–12 mm long, orange intensely curved towards tip about 180°	8–8.5 mm long, orange-red straight	ca. 15 mm long, yellow erect
Anther	ca. 5 × 2 mm, orange	ca. 6.5 × 2.5 mm, orange-red	ca. 7 mm long, bright-yellow
Connective	red	orange-red	bright-yellow
Ovary	ca. 2.5 mm diameter densely villous	ca. 2 mm diameter glabrous	ca. 3 mm diameter densely villous
Stigma	ca. 0.5 mm wide, dark red	ca. 1 mm wide, yellow	ca. 1 mm wide, green
Style	filiform, creamy white dark red tinged towards stigma	filiform, white yellow tinged towards stigma	filiform, white green tinged towards stigma

## Supplementary Material

XML Treatment for Hedychium putaoense
